# Identification of the critical residues responsible for differential reactivation of the triosephosphate isomerases of two trypanosomes

**DOI:** 10.1098/rsob.160161

**Published:** 2016-10-12

**Authors:** Monica Rodríguez-Bolaños, Nallely Cabrera, Ruy Perez-Montfort

**Affiliations:** Departamento de Bioquímica y Biología Estructural, Instituto de Fisiología Celular, Universidad Nacional Autónoma de México, Av. Universidad 3000, Coyoacán, 04510 México DF, México

**Keywords:** triosephosphate isomerase, critical residues, reactivation, protein folding, guanidine hydrochloride, site-directed mutagenesis

## Abstract

The reactivation of triosephosphate isomerase (TIM) from unfolded monomers induced by guanidine hydrochloride involves different amino acids of its sequence in different stages of protein refolding. We describe a systematic mutagenesis method to find critical residues for certain physico-chemical properties of a protein. The two similar TIMs of *Trypanosoma brucei* and *Trypanosoma cruzi* have different reactivation velocities and efficiencies. We used a small number of chimeric enzymes, additive mutants and planned site-directed mutants to produce an enzyme from *T. brucei* with 13 mutations in its sequence, which reactivates fast and efficiently like wild-type (WT) TIM from *T. cruzi*, and another enzyme from *T. cruzi,* with 13 slightly altered mutations, which reactivated slowly and inefficiently like the WT TIM of *T. brucei*. Our method is a shorter alternative to random mutagenesis, saturation mutagenesis or directed evolution to find multiple amino acids critical for certain properties of proteins.

## 1. Introduction

It is generally anticipated that proteins with very similar sequences will also exhibit similar functional and physico-chemical properties. Although this is commonly true, some proteins that have a high sequence identity and great functional and structural similarity can have important quantitative differences in some physico-chemical properties such as protein folding or protein reactivation. Such is the case for the triosephosphate isomerases (TIMs) from *Trypanosoma brucei* and *T. cruzi*. These two trypanosomes diverged around 100 million years before the present [1] and thus many of their proteins have high similarity in their sequences. In the case of their TIMs, they have 73.9% identity and a sequence similarity of 92.4%. The three-dimensional structures for both enzymes have been determined and they superpose with an RMSD of 0.96 Å. TIM is the prototype of the (β/α)_8_ barrel fold family of proteins. It is a homodimer and, even though each monomer contains the three amino acids of the catalytic site—K13, H95 and E167, using the numbering of the sequence of TIM from *T. brucei* (TbTIM)—it is active only in the dimeric form. TbTIM has a sequence of 250 amino acids, and TIM from *T. cruzi* has a sequence of 251 amino acids. For simplification purposes, the numbering of the sequence of TbTIM is used in this work. We have previously reported that TbTIM and TcTIM show quantitative and qualitative differences in their susceptibility to digestion with subtilisin [2] and quantitative differences in their susceptibility to several low molecular weight agents [3], and in particular to sulfhydryl reagents [4–8]. Another quantitative difference has been reported in their velocity and extent of reactivation from guanidine chloride (GdnHCl) unfolded monomers [9].

The denaturation pathway of TIM from several species has been widely studied [10–13]. Several papers describe it as a three-state process [11,13] in which first the dimers dissociate into monomers that have no catalytic activity but retain considerable tertiary structure, and afterwards the monomers unfold completely. The reactivation of TIM from GdnHCl unfolded monomers has also been studied [9,14–16]. In this case, and in particular for TbTIM and TcTIM, the monomers refold upon the removal of the denaturant and subsequently form an intermediate dimer that undergoes internal rearrangements to yield the native and active dimer. This three-state process occurs in both enzymes and despite their great sequence similarity, the reactivation of TcTIM is two to three times faster and approximately 30% more efficient than for TbTIM [9].

In this work, we used a new method, based on the one previously described by García-Torres *et al*. [17], of progressive grafting of different portions of one of these two homologous proteins to the equivalent region of the other protein, to identify the parts of the TIMs that participate in the quantitative differences observed in the reactivation of both proteins. The strategy also used ‘additive’ and systematic site-directed mutagenesis to identify the role of individual amino acids involved in the occurrence, control, extent and speed of the differences in reactivation shown by these two proteins. In the end, we were able to produce an enzyme with 95% of the sequence of TbTIM that had 13 mutations within the first 60 amino acids of that sequence, which had a reactivation behaviour comparable with (and even slightly more efficient than) that of wild-type (WT) TcTIM. We were also able to produce another enzyme with 95% of the sequence of TcTIM with slightly different mutations, also in the first 60 amino acids of its sequence, which had a slow and inefficient reactivation pattern comparable to that of WT TbTIM. Our experiments allowed us to pinpoint all the amino acids that are critical for this quantifiable physico-chemical behaviour.

## 2. Material and methods

### 2.1. Design of the genes of chimeric proteins and production of ‘additive’ mutants

The design of the genes for the chimeric proteins was described in detail by García-Torres *et al*. [17]. Briefly, the DNA sequences X03921 for TbTIM and U53867 for TcTIM were used to construct the chimeric proteins. The sequences of TbTIM and TcTIM were divided into eight regions, roughly encompassing one beta sheet one loop and one alpha helix (for the exact distribution, see figure 1 in [17]). Thus, initially, six chimeric enzymes were constructed. The nomenclature used in this work for the chimeric proteins is as follows: TcTIM1–6;TbTIM7,8 has the sequence of regions 1–6 of TcTIM, and the sequence of regions 7 and 8 of TbTIM; or TcTIM1,2;TbTIM3–8 has the sequence of regions 1 and 2 of TcTIM and the sequence of regions 3–8 of TbTIM, etc. (table 1*a*). The genes, for chimeras TcTIM1–6;TbTIM7,8, TcTIM1–5;TbTIM6–8, TcTIM1–4;TbTIM5–8, TcTIM2–8;TbTIM1, TcTIM1,3–8;TbTIM2 and TcTIM2;TbTIM1,3–8, were synthesized by GenScript (Piscataway, NJ). Other chimeras used in this work, which include TcTIM1–3;TbTIM4–8, TcTIM1,2;TbTIM3–8 and TcTIM1;TbTIM2–8, were made as described by García-Torres *et al*. [17]. The gene of chimeric protein TcTIM3–8;TbTIM1,2 was made with three PCRs using Accuzyme DNA polymerase (Bioline, Taunton, MA). In the first reaction, regions 1 and 2 of the sequence of TbTIM were amplified, using the DNA of WT TbTIM and the sequences of the T7 promoter and the sequence 5′TGC GGC AAT CTG GAA CTT GGG GTG TGA AAG ACG3′ (Rv Tc3–8;Tb1,2) as internal initiator. In the second reaction, the sequences of T7 terminator and 5′TCA CAC CCC AAG TTC CAG ATT GCC GCA3′ (Fw Tc3–8;Tb1,2) were used with the DNA of WT TcTIM as template. Finally, in the third reaction, the products of PCR1 and PCR2 were combined, using the sequences of T7 promoter and T7 terminator as external oligonucleotides.

**Figure 1.**
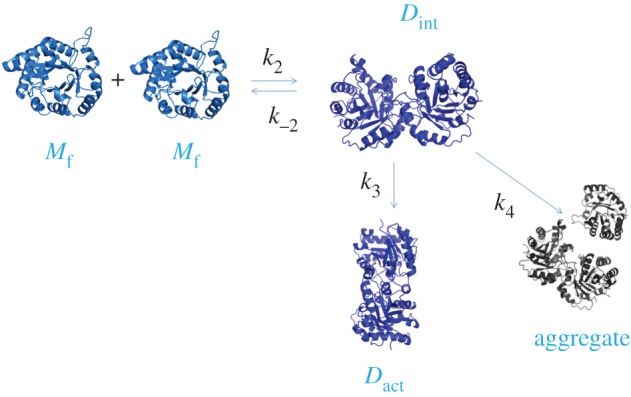
Model of protein folding used to calculate the velocity constants using the program DynaFit. *M* represents the monomer, *D*_int_ represents the intermediate dimer, *D*_act_ represents the active dimer and aggregate represents aggregates.

**Table 1.** (*a*) Diagram of the composition of the chimeric enzymes of TbTIM and TcTIM. The name and the corresponding regions of TbTIM and TcTIM are indicated. In the bar diagram at the right, blue bars show TbTIM regions, and orange bars show TcTIM regions. (*b*) Diagram of the composition of the additive mutants of TbTIM and TcTIM. Additive mutants of region 1 were made using chimera TcTIM 1;TbTIM2–8 as a template. Different amino acids in region 1 were mutated in an additive manner. The dot diagram at the right depicts the 13 different amino acids in region 1; blue dots correspond to TbTIM residues and orange dots correspond to TcTIM residues. For the two site-directed mutants at the bottom of the table chimera TbTIM 1,3–8; TcTIM2 was used as template and the individual mutations of the different amino acids of region 1 are shown. The asterisks indicate that the mutations for the two site-directed mutants are not additive. Blue dots correspond to TbTIM residues and orange dots correspond to TcTIM residues. (*c*) Diagram of the composition of the additive mutants of TbTIM and TcTIM for region 2. Chimera TcTIM 1,3–8;TbTIM2 was used as a template and the seven different amino acids were mutated in an additive manner. The dot diagram at the right depicts the seven different amino acids in region 2; blue dots correspond to TbTIM residues and orange dots correspond to TcTIM residues.

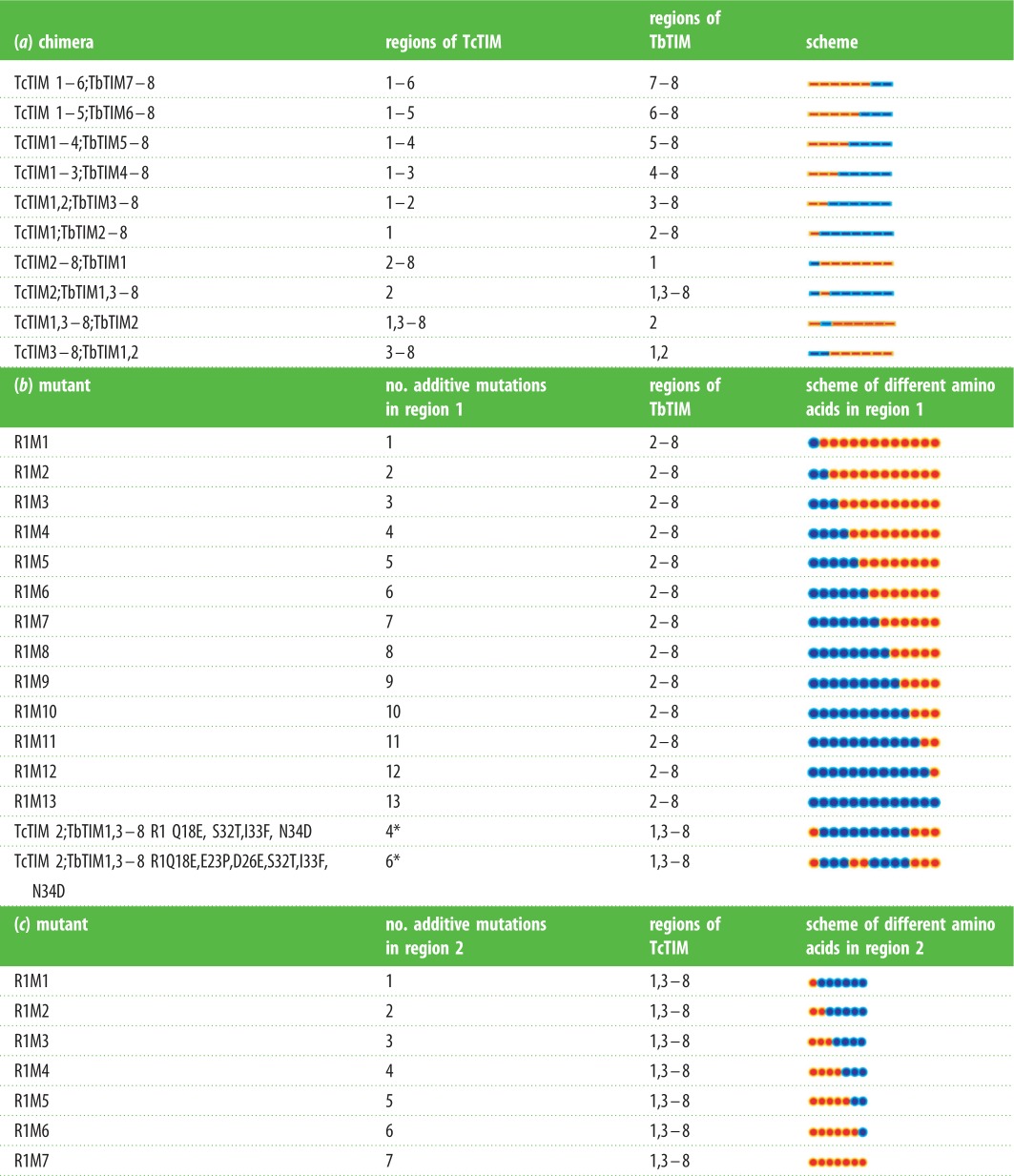

The DNA of two of these chimeras (TcTIM1;TbTIM2–8 and TcTIM1,3–8;TbTIM2) were used as templates for the production of ‘additive’ mutants of regions 1 and 2, respectively (table 1*b*,*c*). These mutants were produced by site-directed mutagenesis using Vent DNA polymerase (Stratagene, CA) and the oligonucleotides listed in table 2.

**Table 2.** Oligonucleotides used for the additive mutagenesis and the site-directed mutagenesis of regions 1 and 2. For additive mutations, the DNA template of the additive mutant immediately preceding it was used.

mutant	FW primer	RV primer
R1M1	5′ TGC AAC GGC TCC CAG AGT TTG CTT GTA 3′	5′ TAC AAG CAA ACT CTG GGA GCC GTT GCA 3′
R1M2	5′ TGC AAC GGC TCC CAG CAG TTG CTT GTA CCA 3′	5′ TGG TAC AAG CAA CTG CTG GGA GCC GTT GCA 3′
R1M3	5′ TGC AAC GGC TCC CAG CAG TCT CTT GTA CCA 3′	5′ TGG TAC AAG AGA CTG CTG GGA GCC GTT GCA 3′
R1M4	5′ TGC AAC GGC TCC CAG CAG TCT TTG TCT GAG CTC ATC GAT ACG CTC 3′	5′ GAG CGT ATC GAT GAG CTC AGA CAA AGA CTG CTG GGA GCC GTT GCA 3′
R1M5	5′ GAG CGT ATC GAT GAG CTC AGA CAA AGA CTG CTG GGA GCC GTT GCA 3′	5′ GAG CGT ATC GAT GAG CTC AGA CAA AGACTG CTG GGA GCC GTT GCA 3′
R1M6	5′ TCT TTG TCT GAG CTC ATT GAT ACG CTC 3′	5′ GAG CGT ATC AAT GAG CTC AGA CAA AGA 3′
R1M7	5′ GAG CTC ATT GAT CTG CTC AAT GCA GCG 3′	5′ CGC TGC ATT GAG CAG ATC AAT GAG CTC 3′
R1M8	5′ CTC ATT GAT CTG TTT AAT GCA GCG ACT 3′	5′ AGT CGC TGC ATT AAA CAG ATC AAT GAG 3′
R1M9	5′ GAT CTG TTT AAT TCC GCG ACT TTT GAT 3′	5′ ATC AAA AGT CGC GGA ATT AAA CAG ATC 3′
R1M10	5′ GAT CTG TTT AAC TCC ACA ACT TTT GAT CAC GAT GTG CAA 3′	5′ TTG CAC ATC GTG ATC AAA AGT TGT GGA GTT AAA CAG ATC 3′
R1M11	5′ GAT CTG TTT AAC TCC ACC AGC TTT GAT CAC GAT GTG CAA 3′	5′ TTG CAC ATC GTG ATC AAA GCT GGT GGA GTT AAA CAG ATC 3′
R1M12	5′ AAC TCC ACA AGC ATC GAT CAC GAT GTG CAA 3′	5′TTG CAC ATC GTG ATC GAT GCT TGT GGA GTT 3′
R1M13	5′ AAC TCC ACA AGC ATC AAC CAC GAT GTG CAA 3′	5′TTG CAC ATC GTG GTT GAT GCT TGT GGA GTT 3′
R1M13ΔALA2	5′ CTT TAA GAA GGA GAT ATA CAT ATG TCC AAG CCA CAA CCC ATC GC 3′	5′ GCG ATG GGT TGT GGC TTG GAC ATA TGT ATA TCT CCT TCT TAA AG 3′
TcTIM2;TbTIM1,3–8 R1Q18E, E23P,D26E,S32T,I33F,N34D	5′ TTT AAC TCC ACA ACC TTT GAT CAC GAC GTG CAA 3′	5′ TTG CAC GTC GTG ATC AAA GGT TGT GGA GTT AAA 3′
TcTIM 2;TbTIM1,3–8 R1Q18E, S32T,I33F,N34D	5′ TTT AAC TCC ACA ACC TTT GAT CAC GAC GTG CAA 3′	5′ TTG CAC GTC GTG ATC AAA GGT TGT GGA GTT AAA 3′
R2M1	5′ GTG CAA TGC GTA GTG GCC CCG ACC TTT GTT CAC CTT GCC ATG 3′	5′ CAT GGC AAG GTG AAC AAA GGT CGG GGC CAC TAC GCA TTG CAC 3′
R2M2	5′ CAA TGC GTA GTG GCC CCG ACC TTT CTG CAC CTT GCC ATG ACC 3′	5′ GGT CAT GGC AAG GTG CAG AAA GGT CGG GGC CAC TAC GCA TTG 3′
R2M3	5′ TGC GTA GTG GCC CCG ACC TTT CTG CAC ATC GCC ATG ACC AAG 3′	5′ CTT GGT CAT GGC GAT GTG CAG AAA GGT CGG GGC CAC TAC GCA 3′
R2M4	5′ GTA GTG GCC CCG ACC TTT CTG CAC ATC CCG ATG ACC AAG GAG 3′	5′ CTC CTT GGT CAT CGG GAT GTG CAG AAA GGT CGG GGC CAC TAC 3′
R2M5	5′ CAC ATC CCA ATG ACG AAG GCG CGT CTT TCA CAC CCC AAA 3′	5′ TTT GGG GTG TGA AAG ACG CGC CTT CGT CAT TGG GAT GTG 3′
R2M6	5′ ACG AAG GCG AGG CTC ACC CAC CCC AAA TTT GTG 3′	5′ CAC AAA TTT GGG GTG GGT GAG CCT CGC CTT CGT 3′
R2M7	5′ ACG AAG GAG CGT CTT ACC AAC CCC AAA TTT GTG ATT GCG 3′	5′ AAT CAC AAA TTT GGG GTT GGT AAG ACG CGC CTT CGT 3′

The nomenclature used for these additive mutants has the general form RXMX, where R is the region in which the mutant occurs and M is the number of additive mutants it has. Thus, mutant R1M1 has one mutation in region 1 and mutant R2M5 has five additive mutations in region 2.

### 2.2. Expression and purification of the chimeric proteins and the mutants

All the genes of the chimeric enzymes and the mutants were cloned into the pET-3a vector (Novagen, WI) with *Nde*I and *Bam*H1 restriction sites, and subsequently sequenced. Each gene was transformed into *Escherichia coli* Bl21-Codon Plus (DE3) RIL cells (Novagen, WI).

Bacteria containing the plasmids with each of the mutant genes were grown in Luria–Bertani medium supplemented with 30 µg ml^−1^ chloramphenicol and 100 µg ml^−1^ ampicillin and were incubated at 37°C until the cell cultures reached an *A*_600_ nm = 0.6. The bacteria were induced with a final concentration of 0.4 mM isopropyl-b-d-thiogalactopyranoside (IPTG), and the cells were incubated 12 h longer at 30°C before harvesting them.

Harvested bacteria were centrifuged for 15 min at 6400*g* and resuspended in 30 ml of lysis buffer (100 mM MES, 1 mM DTT, 0.5 mM EDTA, 0.2 mM PMSF, 300 mM NaCl, pH 6.3). Each suspension was sonicated at a potency of 5 W for 10 times 1 min with 2 min rest between each cycle. The sonicated suspensions were centrifuged at 203 500*g* for 60 min. The supernatant of each chimeric enzyme or mutant was diluted until the final concentration of NaCl was 20 mM and was then applied to a SP Sepharose Fastflow column that had been previously equilibrated with buffer A (50 mM MES pH 6.3). The protein was eluted using a NaCl linear gradient with buffer B (50 mM MES, NaCl 500 mM pH 6.3). Crystalline ammonium sulfate was gradually added up to 70% (w/v) saturation under agitation to the fractions containing TIM. This suspension was further agitated for 16 h and then centrifuged for 15 min at 35 000*g*. The precipitate was resuspended into 3 ml of buffer C (100 mM triethanolamine (TEA), 10 mM EDTA, pH 7.4), and enough ammonium sulfate was added to have a final concentration of 2.2 M. The protein was then applied to a hydrophobic interaction column of butyl toyopearl that had been previously been equilibrated with buffer D (100 mM TEA, 10 mM EDTA and 2 M ammonium sulfate). The protein was eluted with a linear gradient of 2 to 0 M of ammonium sulfate. The fractions containing TIM were pooled and concentrated. Protein concentration was determined at 280 nm, using an extinction coefficient *ɛ* = 34 950 M^−1^ cm^−1^.

There were some differences in the expression and purification of chimeric enzyme TcTIM1,3–8;TbTIM2 and a mutant derived from it: TcTIM1,3–8;TbTIM2(S43P). They were transformed into *E. coli* Origami B (DE3) and were also grown in Luria–Bertani medium supplemented with 100 µg ml^−1^ ampicillin, 34 µg ml^−1^ chloramphenicol, 12.5 µg ml^−1^ tetracycline and 15 µg ml^−1^ kanamycin. These cells were incubated at 37°C until they reached an *A*_600_ nm = 0.6 and then induced with 0.5 mM IPTG. They were further incubated for 24 h at 15°C before harvesting them.

Their purification followed the same steps as those of the other chimeric proteins and mutants until after the cationic exchange column. These proteins were then concentrated and applied to a Superdex 75 gel filtration column previously equilibrated with 100 mM TEA, 10 mM EDTA pH 7.4. The fractions containing protein from this column were concentrated and kept until further use.

### 2.3. Activity assays

Enzyme activity was measured following the conversion of dl-glyceraldehyde 3-phosphate (d,l-GAP) to dihydroxyacetone phosphate using α-glycerolphosphate dehydrogenase (α-GDH) as coupling enzyme at 25°C [17]. The oxidation of NADH was monitored at 340 nm, and the reaction mixture had 10 mM TEA, 10 mM EDTA, 1 mM GAP, 0.2 mM NADH and 20 µg ml^−1^ α-GDH. The reaction was started by adding 5 ng ml^−1^ of the corresponding protein. To calculate kinetic parameters, GAP concentration was varied between 0.05 and 2 mM, and the data were adjusted to the Michaelis–Menten model.

### 2.4. Denaturation and reactivation

The enzymes were denatured by incubation of 0.5 mg of protein ml^−1^ at 25°C for 1 h in 100 mM TEA, 10 mM EDTA, 1 mM DTT and 6 M GdnHCl pH 7.4. For reactivation, aliquots of the latter mixture were diluted 100-fold into a buffer that contained 100 mM TEA, 10 mM EDTA and 1 mM DTT pH 7.4. At this point, the concentration of protein was 5 µg ml^−1^ (183–186 nM). After diluting the proteins, aliquots were removed at different times, and activity was measured in a final volume of 1 ml of reaction mixture. In all cases, the residual concentration of GdnHCl after dilution was 60 mM in the activity assay and 60 µM in the assay mixture. Control experiments in which all enzymes were incubated with 60 mM GdnHCl for the duration of the experiment showed that their activity was not affected. As stated above, activity measurements were performed at 25°C. In cases where reactivation was performed at different concentrations, a range of 10–500 nM monomer was used.

### 2.5. Determination of the rate constants in the reactivation

The rate constants of the reactivation of different chimeras and mutant TIMs were calculated from assays performed at different protein concentrations. Rate constants were calculated in two ways: the first is like the one reported in Zomosa-Signoret *et al.* [9] considering the sequence of reactivation as follows: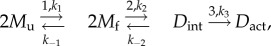;where *M*_u_ represents the unfolded monomer, *M*_f_ the folded monomer, *D*_int_ the intermediate dimer and *D*_act_ the active dimer.

We performed an exponential fit from the plots of reactivation at different concentrations against time, to obtain the pseudo-first-order constants (equation (2.1)) that describe the formation of dimers at different enzyme concentrations2.1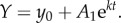;In this equation, *t* represents time, *k* the observed or pseudo-first-order constant, *Y* the catalytic activity, *y*_0_ a value of activity at infinite time and A_1_ represents the amplitude of the curve (activity) at time *t*. The values obtained for the observed constants were plotted against the protein concentration to get the second-order constants or the constants associated with the formation of the active dimer. In these cases, monomer concentrations varied between 10 and 500 nM (data not shown).

Additionally, for the calculation of the velocity constants, in particular the mutants of region 2, we used DynaFit software (from BioKin [18]). For this purpose, different models were tested to calculate the velocity constants associated with refolding. The model we chose finally is described in figure 1. This model omits the information regarding the folding of the monomers. This was because in the work of Zomosa-Signoret *et al*. [9] no differences were found in this first step between TbTIM and TcTIM. The new model also proposes an additional step in the folding process, which is aggregation, introducing a new constant *k*_4_. Thus, the definitions of the constants are: *k*_2_ is the association constant of folded monomers, *k*_−2_ is the dissociation constant of folded monomers, *k*_3_ is the constant for the rearrangement from intermediate dimer to active dimer and *k*_4_ is the constant for the formation of aggregates from intermediate dimers.

The value of the constants was obtained from the best fit of the data of reactivation at different concentrations for each one of the enzymes.

### 2.6. Circular dichroism

Circular dichroism spectra were obtained with an AVIV 62 HDS spectropolarimeter (Lakewood, NJ) in a 0.1 mm quartz cell at 25°C. The scanned wavelengths were 190–260 nm. All enzymes (250 µg ml^−1^) were dialysed in phosphate buffer 10 mM, pH 7.4 and were filtered through 0.45 µm membranes. The spectra show the mean of two independent enzyme preparations with three replicas.

## 3. Results

### 3.1. Kinetic characterization and secondary structure of the enzymes

The kinetic parameters and constants were determined for the WT, all chimeric and mutant enzymes, as already stated, in the direction of G3P to DHAP (table 3). In general, the data obtained for the chimeric enzymes and the additive mutants of region 1 are similar to those of the WT enzymes. In the case of the additive mutants of region 2, some parameters showed an important variation, in which the first mutants of this region had very diminished catalytic capacity (e.g. TcTIM1,3–8;TbTIM2 and additive mutant R2M1). The subsequent additive mutants had better catalytic parameters that were similar to those of WT enzymes, starting from additive mutant R2M4.

**Table 3.** Kinetic parameters of the chimeric enzymes, the additive mutants of regions 1 and 2 and of mutants obtained by site-directed mutagenesis. All data shown are the means of three independent determinations.

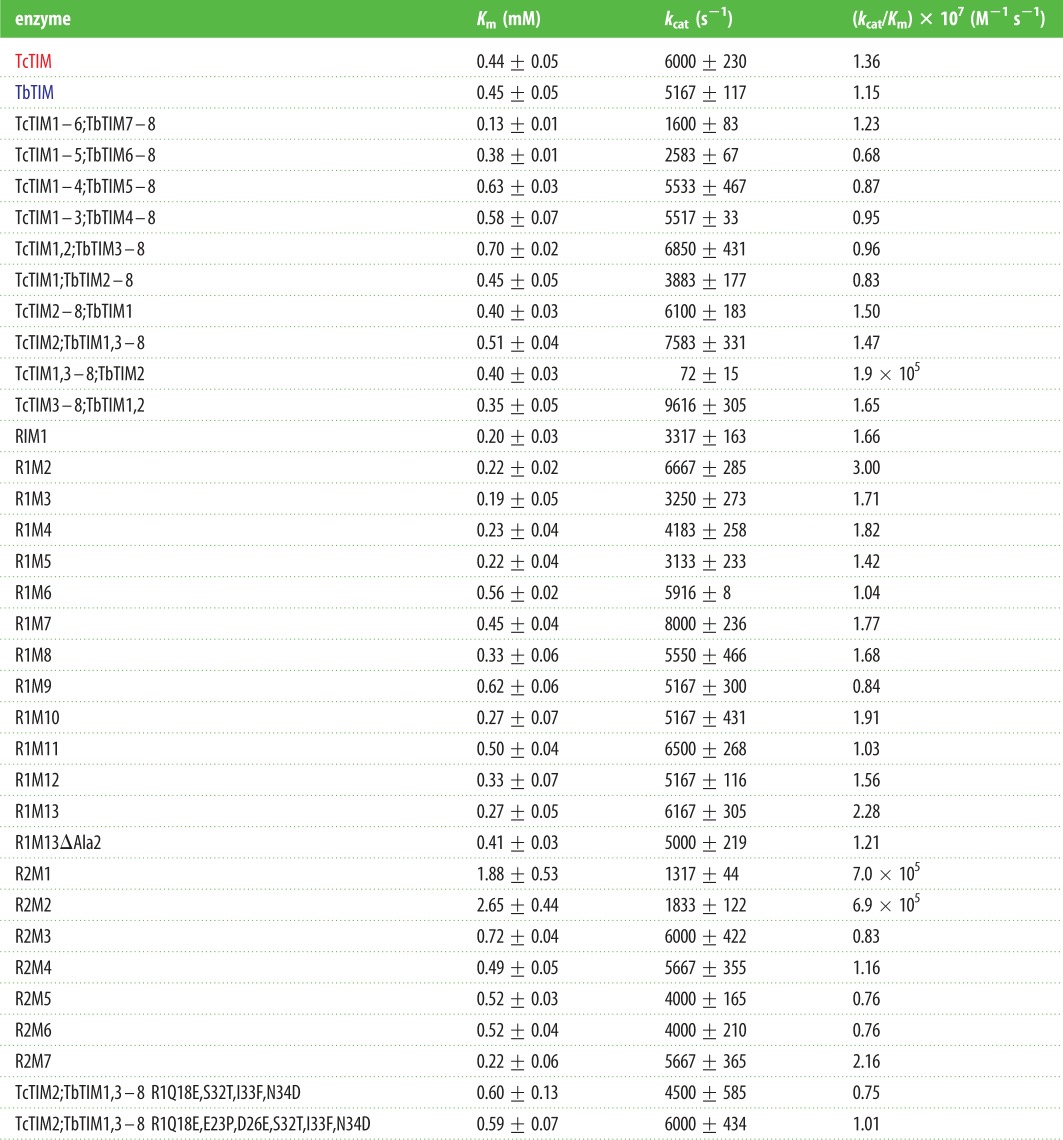

**Figure 2.**
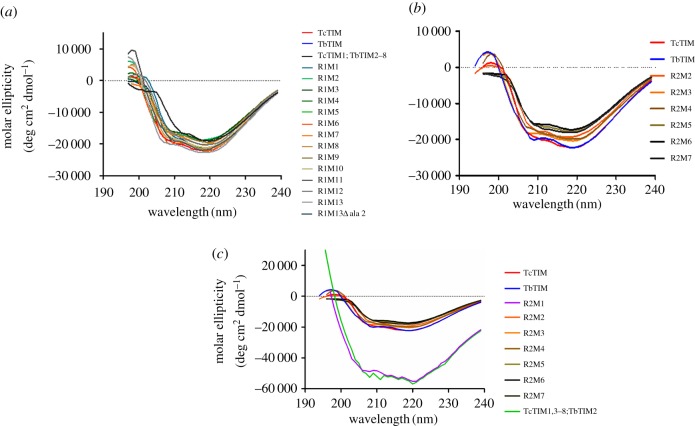
Circular dichroism spectra of chimeric enzymes, additive mutants and mutant enzymes. Proteins were dialysed against phosphate buffer pH 7.4, and spectra were made with 250 µg ml^−1^ of each enzyme in the cell. (*a*) Spectra of additive mutants of region 1. (*b*) Spectra of additive mutants of region 2. (*c*) Comparison of spectra of chimeric enzyme TcTIM1,3–8;TbTIM2 with those of the WT enzymes and additive mutants of region 2.

To assess if there were any important changes in the secondary structure of the enzymes, these were subjected to circular dichroism analysis. The results indicate that they correspond to a structure of a β/α barrel with two negative signals at 222 nm and another between 208 and 201 nm, and a positive signal between 190 and 195 nm. Most mutants also show that their structural profile corresponds to the TIM barrel motif, but they have slight variations in the intensity or form of the signal owing to changes in the mutagenesis [19], which generate small changes in the folding of the enzyme (figure 2). The structures with greatest variation were chimers TcTIM1;TbTIM2–8 and TcTIM1,3–8;TbTIM2. In the case of chimer TcTIM1;TbTIM2–8, the intensity of the signal is diminished for the α-helixes, which could indicate a difference in the folding of this enzyme. Catalytically, it has slightly lower values than the WT enzymes showing it has a perturbation in the catalytic site. In addition, the great majority of the additive mutants in region 1 show a *K*_m_ of about one-half of the *K*_m_ for the WT enzymes indicating that the catalytic site is also affected (table 3). In the case of the chimeric enzyme TcTIM1,3–8;TbTIM 2 and additive mutant R2M1, for which the signals of molar ellipticity are more intense than for the rest of the enzymes (figure 2*c*), the results are discussed below (§4.2).

### 3.2. Regions 1 and 2 are responsible for differences in the reactivation of monomers unfolded with GdnHCl

**Figure 3.**
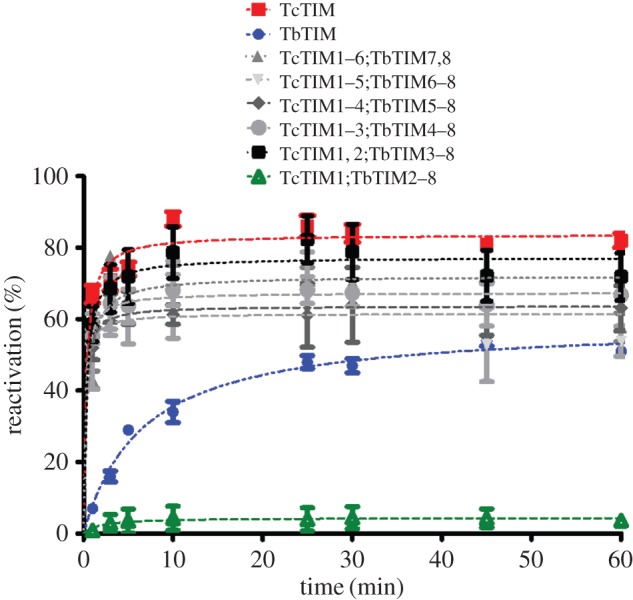
Reactivation of WT TbTIM, WT TcTIM and six chimeric enzymes. Proteins (500 µg ml^−1^) were treated with 6 M GdnHCl for 1 h at 25°C. The enzymes were then diluted 100-fold, and reactivation was measured. Results are expressed as percentage of recovered activity, where 100% is the activity of the native enzymes incubated with the residual concentration of GdnHCl (60 mM).

**Figure 4.**
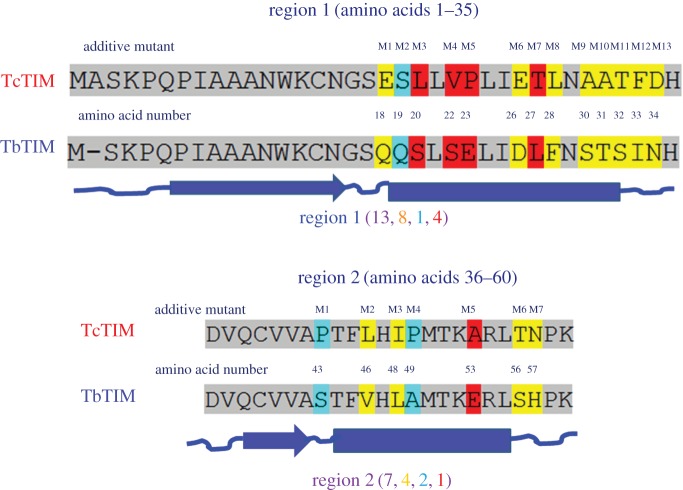
Aligned sequences of regions 1 and 2 of WT TbTIM and WT TcTIM. The differences in the amino acids are highlighted as conservative (similar size and polarity) in yellow, semiconservative (similar polarity) in cyan, and without similarity in red. Secondary structure elements are shown below in blue as lines (loops), arrows (beta sheets) and barrels (alpha helixes).

When the reactivation assays were performed with the chimeric enzymes produced from TbTIM and TcTIM, almost all showed an intermediate behaviour to the one seen with the WT enzymes (figure 3). However, when chimeric enzyme TcTIM1;TbTIM2–8 was tested, the reactivation was extremely poor (figure 3, green triangles) and very slow (approx. only 5% of the reactivation) when compared with chimeric enzyme TcTIM1,2;TbTIM2–8, whose reactivation was as fast and efficient as that of WT TcTIM (figure 3, black squares). These results meant that the different amino acids in regions 1 and 2 between the sequences of WT TbTIM and WT TcTIM were responsible for the changes observed in reactivation. These two regions have 13 and 7 differences, respectively (from a total of 20; figure 4). To ascertain that these two regions were indeed implicated in some aspect of the process of reactivation, we made a new set of chimeric enzymes with all possible combinations of regions 1 and 2.

### 3.3. Reactivation of chimeric enzymes of regions 1 and 2

**Figure 5.**
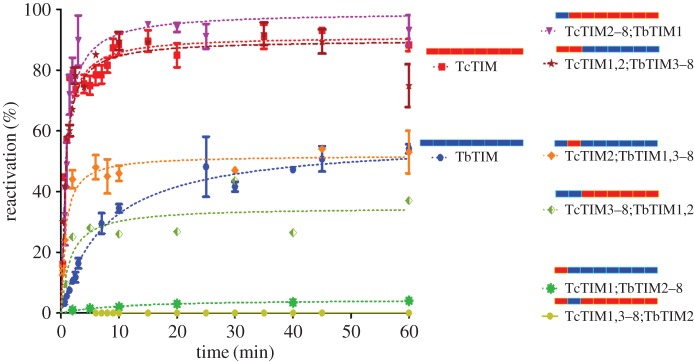
Reactivation of WT TbTIM, WT TcTIM and all the chimeric enzymes of regions 1 and 2. The cartoons with bars at the right represent the regions of TbTIM (blue) and TcTIM (orange) for each enzyme. Proteins (500 µg ml^−1^) were treated with 6 M GdnHCl for 1 h at 25°C. The enzymes were then diluted 100-fold and reactivation was measured. Results are expressed as percentage of recovered activity, where 100% is the activity of the native enzymes incubated with the residual concentration of GdnHCl (60 mM).

The six chimeric enzymes with all possible combinations of regions 1 and 2 were assayed and the results are shown in figure 5. To test region 1, we constructed chimeric enzymes TcTIM1;TbTIM2–8 and TcTIM2–8;TbTIM1. Chimeric enzyme TcTIM1;TbTIM2–8 reactivated minimally and very slowly, whereas chimeric enzyme TcTIM2–8;TbTIM1 was 100% efficient (even better than WT TcTIM) and also very fast. On the other hand, to test region 2, we constructed chimeric enzymes TcTIM2;TbTIM1,3–8 and TcTIM1,3–8;TbTIM2. These enzymes showed two distinct behaviours, the first was shown by chimeric enzyme TcTIM2;TbTIM1,3–8, which reactivated just as fast as WT TcTIM but only partially, to the extent that TbTIM does (40%); the second was that chimeric enzyme TcTIM1,3–8;TbTIM2 did not reactivate at all.

When the remaining combinations of both regions were assayed, chimeric enzyme TcTIM1,2;TbTIM3–8 reactivated in a similar manner to WT TcTIM (90%), whereas chimeric enzyme TcTIM3–8;TbTIM1,2 reactivated somewhat less than WT TbTIM and also, like this last enzyme, did it slowly. It should be pointed out that chimeric enzyme TcTIM3–8;TbTIM1,2 was unstable and tended to dissociate at the concentration and the times used in the assays, which is why, to make its results comparable to the other proteins, the results shown for this enzyme in figure 5 are normalized against a control that did not receive the usual incubation of 1 h at 25°C.

### 3.4. Synergistic and antagonistic combinations of regions 1 and 2

As can be seen in figure 5, there are combinations of regions 1 and 2 that favour or hinder reactivation. The combination that produces an extremely inefficient or null reactivation is region 1 of TcTIM and region 2 of TbTIM; this combination is present in chimeric enzymes TcTIM1;TbTIM2–8 (which only reaches 5% reactivation) and TcTIM 1,3–8;TbTIM2 (which does not reactivate). The combination that favours reactivation is region 1 of TbTIM and region 2 of TcTIM; in this case, reactivation occurs in a fast and efficient manner such as with chimeric enzymes TcTIM2–8;TbTIM1 (100%) and TcTIM2;TbTIM1,3–8 (90%).

### 3.5. Velocity constants of chimeric enzymes of regions 1 and 2

The limiting steps in reactivation of TIM are the association of the monomers and the inner rearrangement that transforms the intermediate dimer into the active dimer [9]. To obtain the velocity constants of these two steps, we reactivated chimeric enzymes of regions 1 and 2 at different concentrations that varied from 10 to 500 nM of monomer. In all cases, excepting chimeric enzyme TcTIM3–8;TbTIM1,2, the plot of absorbance versus time was linear, indicating that the reactivation was not affected by the dissociation of the enzyme during the measurement of the activity (data not shown). Table 4 shows the values of the velocity constants determined for the chimeric enzymes of regions 1 and 2, except for chimeric enzymes TcTIM1,3–8;TbTIM2 and TcTIM3–8;TbTIM1,2, for which no data could be obtained. It should be noted that the velocity constants that are related to the association of monomers were always equal to or larger than that obtained for WT TcTIM in those proteins that have region 2 of TcTIM. On the contrary, when the proteins have region 2 of TbTIM, the constants are lower, even by one order of magnitude, than those reported for the WT enzymes.

**Table 4.** Calculated velocity constants for additive mutants of region 2 and for some chimeric enzymes.

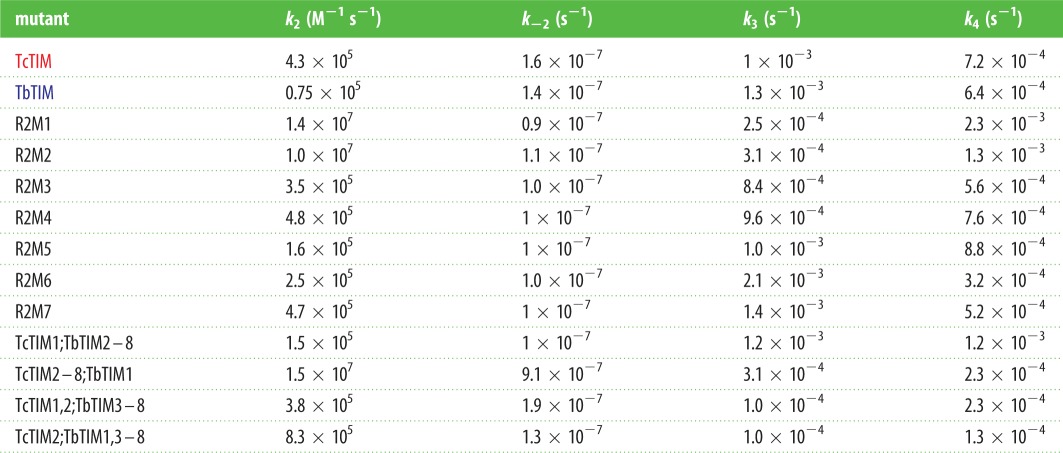

### 3.6. Reactivation of the additive mutants of region 1

To continue locating the amino acids that are important for reactivation in these two regions, a group of additive mutants was prepared with each, and all the residues that are different in region 1 between WT TcTIM and WT TbTIM, by the progressive and cumulative site-directed mutagenesis converting chimeric enzyme TcTIM;TbTIM2–8 into WT TbTIM. This involved the production of 13 additive mutants.

**Figure 6.**
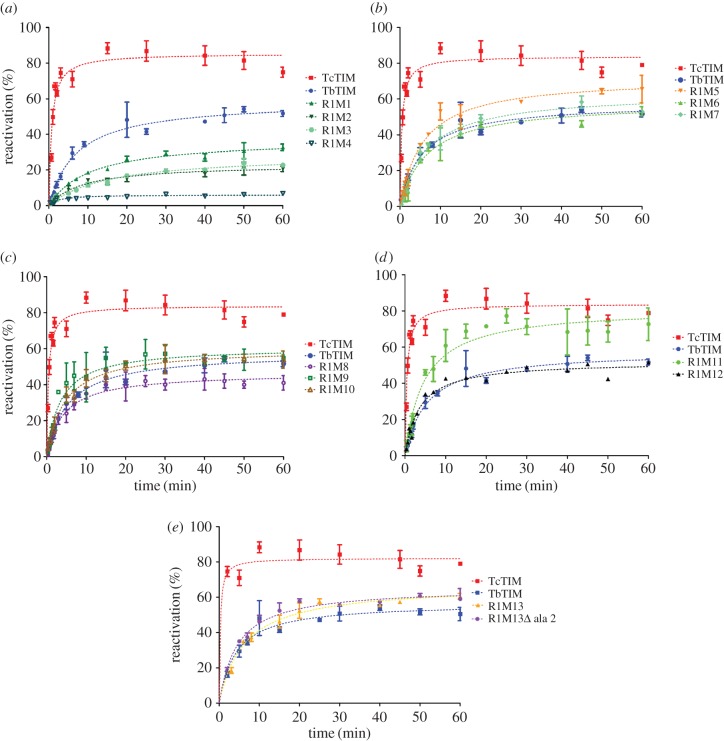
(*a*–*e*) Reactivation of WT TbTIM (blue), WT TcTIM (red) and all the additive mutants of region 1. Proteins (500 µg ml^−1^) were treated with 6 M GdnHCl for 1 h at 25°C. The enzymes were then diluted 100-fold, and reactivation was measured. Results are expressed as percentage of recovered activity, where 100% is the activity of the native enzymes incubated with the residual concentration of GdnHCl (60 mM). The data shown are the mean of three separate and independent experiments.

In the reactivation kinetics of these additive mutants of region 1 (figure 6), we observed that when the first mutation was introduced (R1M1) the reactivation was about 50% as efficient as that of WT TbTIM. Two further additive mutations R1M2 and R1M3 had the effect of reducing activation efficiency by approximately 10%. The fourth additive mutant R1M4 showed an important diminishment in its reactivation capacity with a behaviour similar to chimeric enzyme TcTIM1;TbTM2–8. However, when the fifth additive mutant R1M5 was assayed, the reactivation of this enzyme was approximately 10% more efficient than WT TbTIM. The sixth additive mutant R1M6 returned the reactivation pattern to one like that of WT TbTIM and the seventh additive mutant R1M7 has no apparent effect on the reactivation. It is important to point out here that an additive mutation may have either positive (R1M1, R1M5), negative (R1M2, R1M4, R1M6) or neutral (R1M3, R1M7) effect on the reactivation pattern when compared with the additive mutant that preceded it. The eighth additive mutant showed a decrease in reactivation capacity of approximately 10% with respect to R1M7. However, the ninth additive mutant R1M9 not only increased the velocity of reactivation, improving WT TbTIM, but also did it with a greater efficiency, at least for the first 10 min of the kinetic analysis. The 10th additive mutant R1M10 returns to a reactivation pattern like that of WT TbTIM, but the 11th additive mutant R1M11 again increases the efficiency of the reactivation by approximately 20% over that of WT TbTIM. Both the 12th and 13th additive mutants, R1M12 and R1M13, respectively, return the reactivation pattern to that of WT TbTIM. Because the sequence of WT TcTIM has an additional Ala in position 2, when compared with the sequence of WT TbTIM, we constructed an additive mutant that lacked this Ala in its sequence to test the possibility that this additional amino acid might have some influence on the reactivation pattern. Additive mutant R1M13ΔAla2 had an equal reactivation pattern to R1M13 (figure 6).

### 3.7. Structural analysis of the relevant amino acids in region 1

Trying to understand why these mutations had the observed effect on the reactivation patterns observed, we performed a search of the contacts with other residues at a distance of 4 Å from the mutated amino acids, as well as of the mutated amino acids themselves in the structures with PDB identifiers: 5TIM (TbTIM) and 1TCD (TcTIM). All changes in region 1 are in helix 1 and the beginning of the loop that connects region 1 with region 2. The external face of helix 1 is exposed to the solvent and interacts with helices 2 and 8 on both sides, whereas the internal face interacts with beta sheet 1. It was observed in previous studies that TcTIM has a looser conformation than TbTIM at this site, with fewer interactions between these regions [5]. One of the amino acids that produce this structural difference is Pro 23, which is in the middle of the helix and exposed to solvent, generating a wider gap in the turns of the helix (approx. 1.2 Å). This opening not only decreases the contacts of the residues in this helix, but also modifies the spatial orientation of other amino acids such as Glu 18 (which corresponds to Gln 18 in TbTIM).

In our results of the mutagenesis of region 1, the change of the amino acid in position 18 (Glu for Gln) has one of the strongest effects even though it is quite conservative in both the size and the form of the residue. The important changes in the reactivation of chimeric enzyme TcTIM1;TbTIM2–8 generated by mutating this amino acid are probably caused by the change in orientation of this residue owing to the presence or absence of Pro 23. The structure of TbTIM (without Pro 23) shows a helix 1 that is more compact and with more interactions, allowing Gln 18 to have an orientation that permits the formation of a hydrogen bond with Asp 85 of region 3, monomer B. The native Glu 18 of TcTIM has an orientation that does not permit this interaction (see figure 3 in [5]). Thus, these observations point to the importance of the interactions between the amino acids in positions 18 and 23 in the sequences of TbTIM and TcTIM, and provide a structural explanation for our experimental results.

One question that arose was why additive mutant R1M1 had such a drastic change in reactivation behaviour given that, even though amino acid 18 was mutated, Pro 23 was still present.

Because chimeric enzyme TcTIM1;TbTIM2–8 has mainly the sequence of TbTIM, which structurally has more contacts between regions 1 and 2 than TcTIM, it could be possible that the mutation produced a rearrangement of the amino acid in position 18, in which perhaps one of the two contacts with region 3 of monomer B was restored. Because no crystal structure of additive mutant R1M1 is available, we performed homology modelling, using the Swiss Model server and the coordinates of both WT TIMs [20–22]. The server produced two models in which the amino acid in position 18 is in an intermediate position from the ones observed in WT TbTIM and TcTIM. In this case, one of the two hydrogen bonds was restored, and this should explain the big change in the reactivation behaviour of additive mutant R1M1. This suggests that this amino acid is related to the association between subunits.

**Figure 7.**
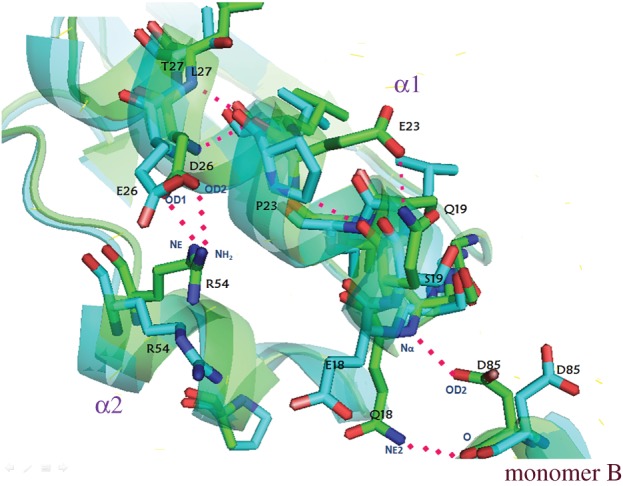
Superposition of the three-dimensional structures of regions 1 of WT TbTIM (green) and WT TcTIM (cyan). In TcTIM, the presence of P23 in the middle of helix 1 produces an aperture of 1.2 Å in the turns of this helix that results in additional interactions with neighbouring residues, which TbTIM does not have. This results in the different conformation of Q18 and E18 in TbTIM and TcTIM, respectively, preventing the latter from forming a contact with monomer B. In alpha-helix 2 (α2) R54 in the structure of TbTIM is able to form a contact with D26, which is not the case for TcTIM.

When Pro 23 was mutated, we saw that the reactivation increased greatly in efficiency; it was even better than TbTIM. This is due to the rearrangement of the helix (changing the residue stabilizes the helix and restores the interactions with other amino acids), yet it is not until the mutagenesis of the amino acid at position 26 (R1M6) when the reactivation behaviour is again like that of WT TbTIM. This is a conservative change again in size and form of the amino acid, but, like the change of the amino acid in position 18, it modifies the charge. Observing the close contacts of amino acids in positions 23 and 26, these interact both with Arg 54 of region 2, which is the same residue in both TbTIM and TcTIM, but which has a different orientation in the crystal structures of the WT enzymes (figure 7). Arg 54 in TbTIM is oriented in a manner that permits the formation of a hydrogen bond with Asp26, whereas this interaction is absent in TcTIM.

We do not know how these amino acids interact in the early stages of folding during reactivation, but it is clear that they are important to guide the process of determining the accessible conformational space for these enzymes.

Even though our statements regarding the changes in position of the amino acids mentioned previously were made comparing some available crystallographic structures of TIMs, the lack of the structures of the enzymes we studied, in particular, makes the movements we propose speculative. However, there are previous works in which the mutation of some residues result in important changes for a certain function [19]. As we could observe in solution (figure 2), these mutants show differences in their secondary structure, indicating that mutagenesis changes the number of contacts in the enzyme, so that it is possible that the variation of the reactivation and folding patterns could be due to the correct or incorrect positioning of one or more key residues.

### 3.8. Reactivation of the additive mutants of region 2

**Figure 8.**
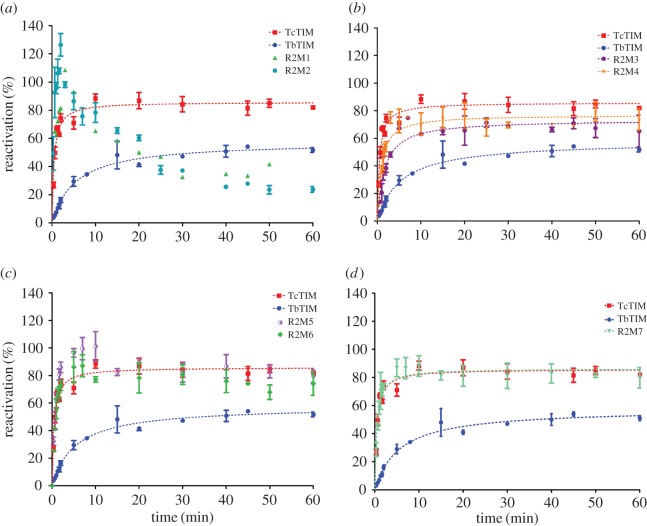
(*a*–*d*) Reactivation of WT TbTIM (blue), WT TcTIM (red) and all the additive mutants of region 2. Proteins (500 µg ml^−1^) were treated with 6 M GdnHCl for 1 h at 25°C. The enzymes were then diluted 100-fold and reactivation was measured. Results are expressed as percentage of recovered activity, where 100% is the activity of the native enzymes incubated with the residual concentration of GdnHCl (60 mM). The data shown are the mean of three separate and independent experiments.

The additive mutants to analyse region 2 were built using chimeric enzyme TcTIM1;3–8;TbTIM2 as the template. In this case, there are seven differences in the sequences of region 2, and the purpose was to gradually transform chimeric enzyme TcTIM1;3–8;TbTIM2 into the sequence of WT TcTIM. To our surprise, in the reactivation kinetics of additive mutants R2M1 and R2M2, a new and very different behaviour was observed (figure 8). Initially, during the first 10 min, the reactivation values rise extremely fast and reach values that exceed 100% reactivation. The curve then falls and reaches reactivation efficiencies similar to or somewhat below the values obtained for TbTIM. The reactivation curve has again a similar form: biphasic, with an increase in activity in the fast phase and an activity decrease in the slow phase. When the third additive mutation is introduced (R2M3), it still has a peak of highest activity approximately at 10 min (which now reaches around 80% reactivation), and then follows intermediate values situated in an intermediate position between those of the WT enzymes. This additive mutant reactivates with greater velocity than WT TbTIM, but is slower than WT TcTIM, and its maximum reactivation efficiency is approximately 15% lower than that of WT TcTIM. The fourth additive mutant (R2M4) is very similar to the third but is slightly faster and more efficient. In the case of the fifth additive mutant R2M5, the final reactivation efficiency is the same as that of WT TcTIM but it still shows a peak with approximately 100% reactivation at 10 min. The sixth additive mutant R2M6 shows a very similar behaviour to R2M5 and the seventh additive mutant R2M7 shows the same reactivation pattern as WT TcTIM (without the initial peak). Table 4 shows the velocity constants obtained for these additive mutants.

Mutagenesis of region 2 revealed that two aspects of refolding were affected. The first was the speed with which the refolding occurs and the second was the process of aggregation. As was mentioned previously, the limiting steps in reactivation are the association of monomers and the internal rearrangement going from intermediate dimer to native dimer. Region 2 has several interface residues (Val 46, Leu 48 and Ala 49 in TbTIM or Leu 46, Ile 48 and Pro 49 in TcTIM) that interact with the other monomer, which naturally will affect the association when they are modified.

Calculating the velocity constants of reactivation of mutant enzymes of these amino acids allowed us to see which points of the process were affected and which residue was responsible for each effect (table 4).

Some of the residues that are crucial for the reactivation of chimeric enzyme TcTIM1,3–8;TbTIM2 were those in positions 43 and 46 (additive mutants R2M1 and R2M2). These mutations generated a different behaviour from that seen before for any enzyme, yet without modifying these residues, the reactivation of chimeric enzyme TcTIM1,3–8;TbTIM2 would not occur. These amino acids are in the loop that joins beta strand 2 with helix 2, and all of them are very close to the catalytic loop.

The variation of the kinetic constants in these two additive mutants indicates that there must be a folding intermediate with catalytic activity that produces the peak (figure 8*a*). Constants *k*_2_ and *k*_3_ in these mutants are much higher, when compared with the WT enzymes. An increase in the association velocity of the monomers generates the accumulation of the folding intermediate with activity, whereas the subsequent fall that is observed could be due to aggregation of the enzymes, because the constants *k*_4_ are also elevated.

In the case of additive mutants R2M3 and R2M4, the reactivation curve has the same biphasic form, like the rest of the mutants, yet these curves also have a small maximum peak of reactivation during the first 10 min. The values of the *k*_2_ constants are within the range of those reported for the WT enzymes, and for additive mutant R2M4, it is slightly greater, yet the constants *k*_3_ are even higher than those of the WT enzymes, indicating that for these additive mutants both the association of the monomers and the formation of the active dimers are quite important. The reactivation of additive mutant R2M5 already has similar values to those of WT TcTIM, but it still also shows a very small maximum at 10 min, with the other constants similar to those of the WT enzymes, and aggregation starting to play a role. Finally, additive mutant R2M7 has a reactivation behaviour like a WT enzyme in which the peak at 10 min disappears, and the velocity constants are very similar to those of the WT enzymes.

From these results, we conclude that the amino acids of region 2 that favour the association of TIM are those of the first (R2M1) and second (R2M2) additive mutants (positions 43 and 49, respectively) and to a lesser degree those in positions 56 and 57. On the other hand, the conversion of an intermediate dimer with activity to an active dimer is affected by the amino acids in positions 43, 46, 48, 49 and 53, increasing the value of constants *k*_4_ and *k*_3_, an effect that diminishes when amino acids in positions 56 and 57 are mutated, returning the values of these constants to those shown by the WT enzymes.

### 3.9. Site-directed mutagenesis of selected amino acids from regions 1 and 2

**Figure 9.**
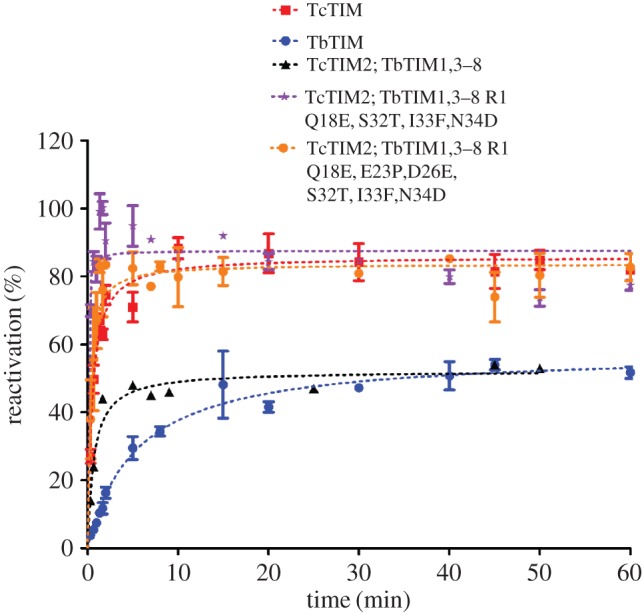
Reactivation of WT TbTIM, WT TcTIM, a chimeric enzyme and two mutants obtained by site-directed mutagenesis. Proteins (500 µg ml^−1^) were treated with 6 M GdnHCl for 1 h at 25°C. The enzymes were then diluted 100-fold and reactivation was measured. Results are expressed as percentage of recovered activity, where 100% is the activity of the native enzymes incubated with the residual concentration of GdnHCl (60 mM). The data shown are the means of three separate and independent experiments.

Having identified the amino acids in regions 1 and 2 that are important for the quantitative change of behaviour in reactivation for TbTIM and TcTIM, we decided to make a mutant enzyme mostly with the sequence of TbTIM, only changing the six amino acids identified in region 1 and the seven amino acids in region 2 from TcTIM, to see if this enzyme would reactivate like TcTIM. The DNA template used to make this mutant enzyme was that of chimeric enzyme TcTIM2;TbTIM 1,3–8, which already has the seven differences in region 2 and has a reactivation pattern similar to TbTIM (figure 9). Mutant TcTIM2;TbTIM1,3–8 R1:Q18E,E23P,D26E,S32T,I33F,N34D was constructed, and, as expected, it had a reactivation pattern just like WT TcTIM (figure 9). The kinetic parameters turned out to be slightly different, because they have a greater affinity for its substrate, but their values are within the range of the WT enzymes (table 3).

As seen with the reactivation of the additive mutants of region 2, all seven amino acids are important, but in the case of the reactivation of the additive mutants of region 1, there were three amino acids that had a greater effect. Two additional mutants were made to study the effects of these amino acids. The mutants were TcTIM2;TbTIM1,3–8 R1:Q18E, S32T,I33F,N34D and TcTIM 2;TbTIM1,3–8 R1:Q18E,E23P,D26E,S32T,I33F,N34D. The first mutant was constructed to explore amino acids at positions 18 and 32. Amino acids at positions 33 and 34 were also changed, because the contact map of these residues show hydrogen bonds in the structure of WT TbTIM, which is absent for residue 32 in the structure of WT TcTIM. The second mutant explored the combination of amino acids at positions 18, 23 and 32, and amino acid at position 26 was added, because it is important for recovering the reactivation pattern of the WT enzyme (see reactivation of the additive mutants of region 1).

As mentioned earlier, chimeric enzyme TcTIM2;TbTIM1,3–8 reactivates rapidly and with an efficiency like that of WT TbTIM (figure 9), but changing amino acids at positions 18, 32, 33 and 34 increased the efficiency of reactivation greatly, making it even better than that of WT TcTIM. This mutant also showed a peak of increased activation at 10 min (like that of the additive mutants of region 2), but at the end of the time in which reactivation was followed (40–80 min) it had lower values, by approximately 10%, when compared with WT TcTIM at the same times (figure 9). This fall at the end of the reactivation period for this mutant can be explained by the interaction that amino acids at positions 32, 33 and 34 have with those at positions 56 and 57 (which are in region 2). In WT TbTIM and WT TcTIM, exactly all of these amino acids are different, so, when they are changed, all interactions, hydrogen bonds, etc., are also altered. Additionally, as already seen in the reactivation of additive mutant of region 2, amino acids at positions 56 and 57 recover the reactivation patterns to that of the WT enzymes and affect the internal rearrangement of the active intermediate and the aggregation. Thus, a perturbation in these sites could produce this behaviour. As in the additive mutants of region 2, the reactivation curve shows a peak of higher reactivation at 10 min, and it appears when the amino acids in positions 43 and 46 are mutated (Ser for Pro and Val for Leu, respectively). The Pro in position 43 changes the contacts between several amino acids in region 1 and region 2, modifying the stability of the enzyme.

## 4. Discussion

### 4.1. The role of the loops in reactivation: region 1

The amino acid sequences of WT TbTIM and WT TcTIM are very similar as are their three-dimensional structures, both have a quantitatively different behaviour in the reactivation of their monomers that have been unfolded with GdnHCl. In this work, we investigated which regions and which amino acids are responsible for these differences. This investigation was based on previous observations that have shown that in TIM only native dimers have catalytic activity and this is considered to be evidence that the quaternary structure of the enzyme is correct [8,9,23,24]. Even though there are also reports of activities in monomeric TIMs, these activities are very much lower than those reported for TbTIM and TcTIM [25,26].

Our results with the additive mutants of region 1 indicate that the amino acids in positions 18, 19, 20, 22, 23 and 26 of the sequence are those that influence reactivation positively to produce the folding pattern of TbTIM, whereas in region 2, all seven amino acids are important for reactivation. In the three-dimensional structure of the enzymes, most of these residues are exposed to the solvent or belong to one of the loops of the TIM barrel. The first, at position 18, is at the beginning of helix 1, just after interfacial loop 1 (which joins beta sheet 1 with helix 1), whereas the amino acids at positions 32, 33 and 34 are in external loop 1, which connects helix 1 with beta sheet 2. In the cases of region 2, residues at positions 43 and 46 (which affect monomer association) are in interfacial loop 2 and amino acids at positions 56 and 57 are in external loop 2. Several studies have shown that loops play an important role in protein folding, because the contacts that they form with the rest of the polypeptide chain limit the available conformational space, accelerating or slowing down the process [27–30]. Thus, the position of these amino acids in the loops could be a factor that guides the folding in these proteins and the difference in their reactivation could be due to the interactions formed in the initial points of folding or to the dihedral angles formed by the side chains of these amino acids. TcTIM has different dihedral angles from TbTIM, because in both enzymes, helix 1 and helix 2 have a Pro in the middle of the sequence, which alters their structure. In contrast with other studies in which the isomerization of Pro is a factor that slows the folding process of proteins [31,32], this does not happen with TcTIM, whose reactivation is faster and more efficient and shows less aggregation than TbTIM.

The interface of TIM is made mainly by some of its loops. In advanced stages of folding, the movement of these loops is a factor that influences the process because they are related to the dimerization of the monomers, one of the crucial steps of folding. The association of the monomers in TIM permits the recovery of optimal catalytic activity [9].

The loops that are involved in dimerization are interfacial loops 1–4 (those that join the beta sheet with the subsequent alpha helix). Loop 3 is the longest loop in the enzyme and it is positioned over loop 2 of the other monomer, allowing the interaction between the residues of interfacial loops 1 and 4. When dimerization takes place the mobility of these loops, together with that of loop 8, is restricted to keep the correct position of the catalytic amino acids. Meanwhile, loops 6 and 7 increase their mobility, so they can continue to protect the catalytic site and allow the entrance of substrates and the liberation of products [28].

Modifying the contacts of loops 1–4 and 8 by mutating amino acids in regions 1 and 2 can increase the mobility of these loops altering the reactivation pattern and the catalytic parameters of the mutant enzymes; the results shown in table 3 and figure 6 are consistent with this.

The *K*_m_ values for the additive mutants from region 1 (R1M1, R1M2, R1M3, R1M4, R1M5, R1M8, R1M9, R1M11 and R1M13) were close to half of the value for the WT enzymes (table 3). This could be due the fact that region 1 contains two of the four catalytic residues of TIM. These are Asn 11 and Lys 13, whose role in catalysis is the recognition of the substrate and the stabilization of the negative charges of the intermediates during the enzymatic reaction [33]. The changes in the amino acid sequence in this region can result in the modification of some contacts of the residues that affect catalysis. Both Asn 11 and Lys 13 are in interfacial loop 1. Several changes in region 1 can modify the interactions and positioning of the rest of the amino acids. The most important residues are in positions 18, 19, 20, 22, 23 and 26, because their changes induce the recovery of the catalytic parameters.

After the association of the monomers, small adjustments in the loops of TIM may follow. That is why in additive mutants of region 1 the change of *K*_m_ to values comparable with those of the WT enzymes occurs when the structure of helix 1 and interfacial loop 1 acquire a conformation like WT TbTIM when Pro 23 and Glu 26 are changed, and the torsion angles of the helix become like those of WT TbTIM (figure 7). This allows the reestablishment of contacts with region 2, reducing mobility and stabilizing this area.

### 4.2. The role of the loops in reactivation: region 2

Region 2 controls the speed of reactivation, possibly because of the close connection it has with region 1 of the same monomer and regions 2 and 3 of the adjacent monomer. Mutants of region 2 had important variations in their kinetic properties because they affect the association of both monomers probably increasing the mobility of the interfacial loops (thus also increasing the catalytic capacity and destabilizing the active site in the enzymes).

Catalysis in chimeric enzyme TcTIM1,3–8;TbTIM2 was strongly affected (table 3). The analysis of its secondary structure by circular dichroism showed the characteristic pattern of a (β/α)_8_ barrel but with a signal considerably more intense than the WT enzymes (figure 2). This has also been previously observed by circular dichroism in equilibrium denaturing studies of TcTIM, where an intermediate with similar characteristics (increase of secondary structure and intensity of fluorescence) was reported [13]. The chimeric enzyme was also analysed by size exclusion chromatography [11] to ascertain that the very low activity was due to the dimer. It had values that were similar to a monoTIM [25,26,34], indicating that the association of the monomers was affected.

Amino acids in positions 43 and 46 in region 2 were found to be critical for the velocity of reactivation (association of the enzyme) and the velocity of aggregation, and they are located at the end of interfacial loop 2 that joins beta strand 2 and helix 2. In additive mutants R2M1 and R2M2, the reactivation curve has a peak with great activity caused by the accumulation of an intermediary species with good activity, which subsequently falls because of the aggregation of this dimeric species during the internal rearrangement to form the native dimer. There are great changes in the velocity constants that affect these phenomena; for example, the association constants for these additive mutants are two orders of magnitude higher than those of the WT enzymes and the aggregation constants are also increased by one order of magnitude, favouring the appearance of the reactive intermediary and its accumulation. On the one hand, the mutation of these two amino acids allows the reactivation of the enzymes, when compared with chimeric enzyme TcTIM1,3–8;TbTIM2, but both the catalytic properties and the kinetic constants of reactivation are affected. These additive mutants also showed a fourfold to sevenfold increase in the value of *K*_m_ over those of the WT enzymes, which means that the mutations favour the association of the monomers, stabilizing the active site. On the other hand, their dissociation constants were slightly smaller than those of the WT enzymes, indicating that the fall in the reactivation pattern after 10 min is not owing to dissociation of the enzyme, but to aggregation during the internal rearrangement to form the native dimer.

The fourth mutation of region 2 (Ala to Pro), just like when a Pro was introduced into a helix when mutating region 1, caused the modification of the dihedral angles with the neighbouring amino acids and the change of contacts with other residues like Arg 54, which has a different conformation in WT TbTIM and WT TcTIM. In this last enzyme, Pro can have more interactions with amino acids in helix 1 (figure 7). The *K*_m_ value of R2M4 is almost equal to that of the WT enzyme.

The amplitude and maximal percentage reached by the reactivation curves and their tendency to remain at stable values (around 60 min) is related to the propensity of each mutant enzyme to aggregate. In region 2, this tendency is associated with amino acids in positions 56 and 57, which are located in external loop 2 near amino acids in positions 32, 33 and 34 of external loop 1. When amino acids in positions 56 and 57 are mutated, the amplitude of the reactivation curve is recovered, and the values at longer times are stabilized, because aggregation is minimal or does not occur. Amino acid in position 57, particularly, makes the initial peak at 10 min disappear (figure 8*d*); it also returns the value of the velocity constant for association to that shown by WT TcTIM, indicating that it affects the association of the enzyme.

In the case of mutant TcTIM2;TbTIM1,3–8 R1:Q18E, S32T,I33F,N34D, the reactivation peak at 10 min appeared again. In this mutant, two of the points (the amino acid in position 18 and the amino acids in positions 32, 33 and 34 together) with greatest effect on the reactivation patterns were changed. Residue 18 is located in internal loop 1, and residues 32, 33 and 34 are located in external loop 1, and, as was previously seen, changes in this external loop have effects on the amplitude of the reactivation curve and the tendency to aggregate, because interactions between loops 1 and 2 are affected. This mutant showed an increase in the amplitude of the reactivation pattern, but it has also a tendency to aggregate, indicating problems in the association between dimers and generating an accumulation of molecular species that is seen as the peak at 10 min (like the one observed for region 2) that eventually leads to aggregation.

Another critical point is the amino acid at position 23. As already mentioned, the introduction of a Pro in the middle of helix 1 produces important changes that affect several contacts all along the helix. In mutant TcTIM2;TbTIM1,3–8 R1:Q18E,E23P,D26E,S32T,I33F,N34D, this residue and the one in position 26 were changed, which allowed a restructuring of helix 1, internal loop 1 and external loop 1, which also allowed a better interaction between helix 1 and helix 2 (figure 7) and a better association of monomers, yielding a reactivation pattern comparable with WT TcTIM.

### 4.3. Aggregation of these enzymes

Chánez-Cárdenas *et al*. [13] reported two critical points where aggregation can occur along the reactivation route for WT TbTIM. The first occurs during the association of the dimers, and results from this work indicate that amino acids in positions 18, 43, 46, 48 and 57 have an effect on this process, because modifying these residues leads to an inefficient reactivation. The second critical point for aggregation is during the restructuring of the dimeric intermediate to the active dimer. Here, amino acids in positions 32, 33, 34, 56 and 57 are those that have an influence, because modifying their interactions influences the amplitude of the reactivation curves, and this is very evident at times near 60 min.

## 5. Conclusion

One of the differences between WT TbTIM and WT TcTIM is the rigidity of their structure. In the case of WT TcTIM, the structure is laxer, which permits a greater degree of conformational freedom and, in the case of reactivation, the environment around regions 1 and 2, favours refolding. From this study, it is clear that the contacts established between these two regions is essential for reactivation to take place.

Our studies have established the critical residues involved in the renaturation/reactivation of two TIMs from two trypanosomatids. The important positions of amino acids in region 1 are 18, 23, 26, 32, 33 and 34, whereas for region 2, they are 43, 46, 48, 49, 53, 56 and 57. To make a TcTIM reactivate with a TbTIM-like behaviour you need to mutate the following amino acids: R1: 18, 19, 20, 22, 23 and 26, and R2: 43, 46, 48, 49, 53, 56 and 57. To make a TbTIM reactivate with a TcTIM-like behaviour you need to mutate amino acids R1: 18, 23, 26, 32, 33 and 34, and R2: 43, 46, 48, 49, 53, 56 and 57. Thus, 13 residues, or 5% of the protein sequence, are critical in determining a specific physico-chemical property of the enzyme.

The amino acids in the internal loops control the process of dimerization, whereas those in the external loops control the stability of the dimer during the transition of intermediate dimer to native dimer, avoiding aggregation.

In this work, we developed a strategy to search systematically multiple amino acids from quite similar proteins that are involved in quantitative differences of their biochemical and/or biophysical properties. We favour this method over a random mutagenesis approach; it is able to find the residues involved in controlling the corresponding properties, with a relatively small and restricted number of chimeric enzymes, additive mutants and planned site-directed mutant proteins, continuously monitored by experimental results (see [17]). Additive mutagenesis provided insights into the process and mechanism of the reactivation of TbTIM and TcTIM. We believe that this experimental approach can be of more general value in studies of protein–function relationships.

## Data accessibility

The data supporting this article have been included in the main text, tables and figures.
