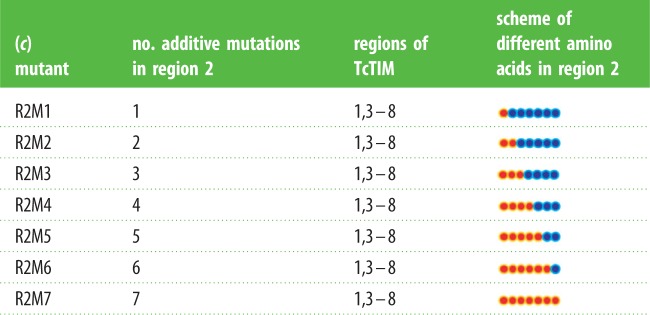# Correction to ‘Identification of the critical residues responsible for differential reactivation of the triosephosphate isomerases of two trypanosomes’

**DOI:** 10.1098/rsob.160294

**Published:** 2016-11-23

**Authors:** Monica Rodríguez-Bolaños, Nallely Cabrera, Ruy Perez-Montfort

*Open Biol.* **6**, 160161. (Published online 12 October 2016). (doi:10.1098/rsob.160161)

In table 1*c*, in the left column, the seven additive mutants of TbTIM and TcTIM for region 2 named R1M1, R1M2, R1M3, R1M4, R1M5, R1M6 and R1M7, should be named R2M1, R2M2, R2M3, R2M4, R2M5, R2M6 and R2M7, respectively.

The correct table appears below

**Table:**